# Natural Chlorophyll-Related Porphyrins and Chlorins for Dye-Sensitized Solar Cells

**DOI:** 10.3390/molecules17044484

**Published:** 2012-04-13

**Authors:** Xiao-Feng Wang, Osamu Kitao

**Affiliations:** 1 Research Center for Organic Electronics, Graduate School of Engineering, Yamagata University, 4-3-16 Jonan, Yonezawa, Yamagata 992-8510, Japan; 2 Energy Technology Research Institute and Research Center for Photovoltaic Technologies, National Institute of Advanced Industrial Science and Technology (AIST), Higashi 1-1-1, Tsukuba, Ibaraki 305-8568, Japan

**Keywords:** porphyrin, chlorin, dye-sensitized solar cell, photosynthesis, organic photovoltaics

## Abstract

Natural-chlorophyll-related porphyrins, including (2H, Zn, Cu)-protoporphyrin IX (Por-1) and Zn-mesoporphyrin IX (Por-2), and chlorins, including chlorin *e*_6_ (Chl-1), chlorin *e*_4_ (Chl-2), and rhodin *G*_7_ (Chl-3), have been used in dye-sensitized solar cells (DSSCs). For porphyrin sensitizers that have vinyl groups at the β-positions, zinc coordinated Por-1 gives the highest solar-energy-to-electricity conversion efficiency (*η*) of up to 2.9%. Replacing the vinyl groups of ZnPor-1 with ethyl groups increases the open-circuit voltage (*V*_oc_) from 0.61 V to 0.66 V, but decreases the short-circuit current (*J*_sc_) from 7.0 mA·cm^−2^ to 6.1 mA·cm^−2^ and the value of *η* to 2.8%. Density functional theory (DFT) and time-dependent DFT (TD-DFT) calculations suggest that the higher *J*_sc_ values of Zn-based porphyrin sensitizers result from the favorable electron injection from the LUMO at higher energy levels. In the case of the chlorin sensitizers, the number of carboxyl protons has a large effect on the photovoltaic performance. Chl-2 with two carboxyl protons gives much higher values of *J*_sc_, *V*_oc_, and *η* than does Chl-1 with three carboxyl protons. Replacing the protons of Chl-1 with sodium ions can substantially improve the photovoltaic performance of Chl-1-based solar cells. Furthermore, the sodium salt of Chl-3 with an aldehyde group at the C7 position shows poorer photovoltaic performance than does the sodium salt of Chl-1 with methyl groups at the C7 position. This is due to the low light-harvesting capability of Chl-3.

## 1. Introduction

Dye-sensitized solar cells (DSSCs) have been commonly regarded as some of the most promising candidates for next-generation photovoltaics [1]. Because of the considerable efforts of many research groups, the current state-of-the-art DSSCs show a solar-energy-to-electricity conversion efficiency (*η*) of up to 11.1% [2]. The development of novel cost-effective dye sensitizers has always been the main focus of DSSC research [3]. The commonly used dye sensitizers in DSSCs are Ru complexes, but this situation is gradually changing, since recent studies have shown that cyclic-tetrapyrrole-based dye sensitizers deliver excellent photovoltaic performance [4,5,6]. On the one hand, the highest obtainable values of *η* for cyclic-tetrapyrrole-based DSSCs are 7.1% for porphyrins [7], 8% for chlorins [8], 6.6% for bacteriochlorins [9], 5.1% for purpurins [10], and 4.5% for phthalocyanines [11]. On the other hand, these cyclic-tetrapyrrole-based molecules are obtained by complicated organic synthetic routes and may not be reproducible in large amounts. In order to minimize the production cost of DSSCs, it is essential to develop inexpensive methods for their consistent large-scale production.

Chlorophylls, the key materials for natural photosynthesis, are the most abundant cyclic-tetrapyrrole-based molecules on the Earth. Today, chlorophyll is produced in large quantities from higher plants and seaweeds and is used extensively in the food industry. From an economic point of view, the best choice may be to fabricate DSSCs with dye sensitizers that can be produced by simple derivatization of natural chlorophyll molecules. 

In the present study, we fabricated DSSCs with commercially available natural-chlorophyll-related porphyrin and chlorin sensitizers. We calculated the molecular orbitals of these sensitizers using density functional theory (DFT) and the absorption spectra from time-dependent DFT (TD-DFT) calculations. We also characterized the photovoltaic performance of DSSCs based on these sensitizers and evaluated in detail the effect of the central metal and the number of protons on porphyrin- and chlorin-based solar cells. 

## 2. Results and Discussion

### 2.1. Porphyrin Sensitizers for DSSCs

**Figure 1:**
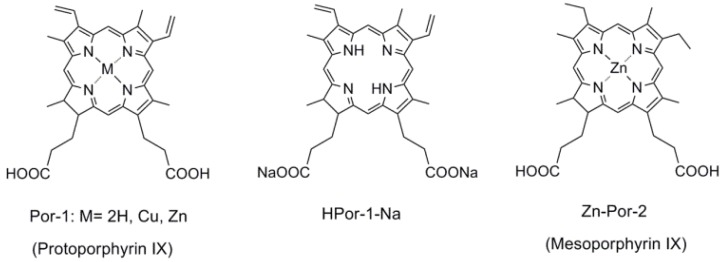
The chemical structures of chlorophyll related porphyrin sensitizers.

Figure 1 shows the chemical structures of the porphyrin sensitizers used in the present study. Por-1 has two ethyl carboxyl groups at the two β-positions of the porphyrin ring and two vinyl groups at the two β-positions on the opposite side. Here, different central metals, including 2H, Zn, and Cu, have been used as the coordinating metal for Por-1. Por-1 with sodium ions has also been used to study the effect of protons, because the binding mode of the dye onto the TiO_2_ surface is a major factor affecting the photovoltaic performance of solar cells [12]. Por-2 is obtained by replacing the vinyl groups of Por-1 by two ethyl groups, and this can lead to a slightly smaller conjugation system that causes Por-2 to be less absorptive in the long-wavelength region.

**Figure 2:**
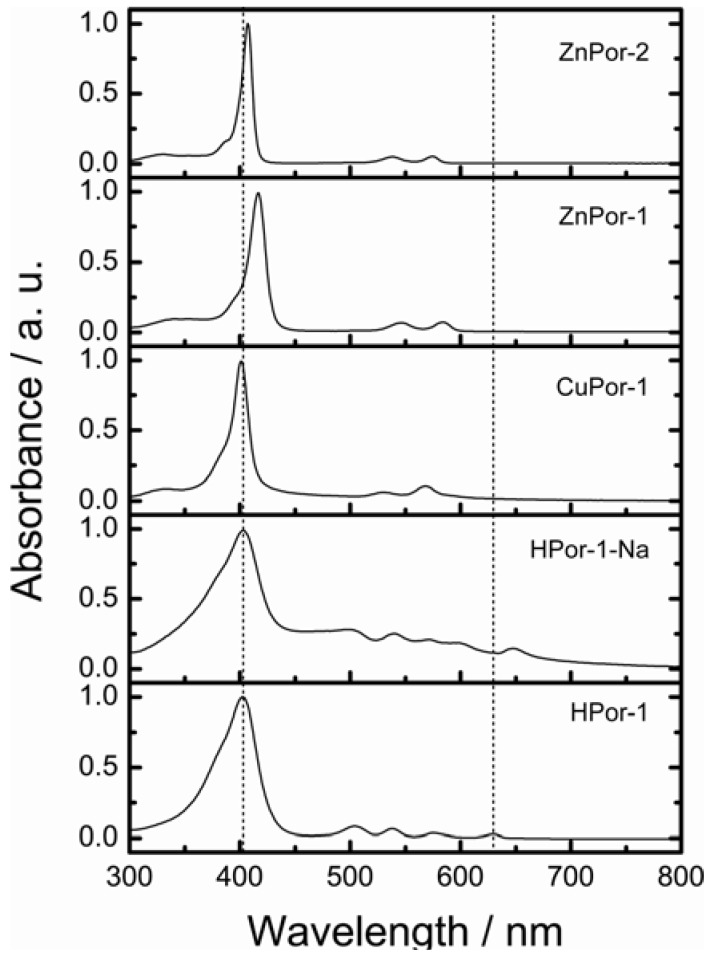
The electronic absorption spectra of porphyrin sensitizers in ethanol solution.

**Figure 3:**
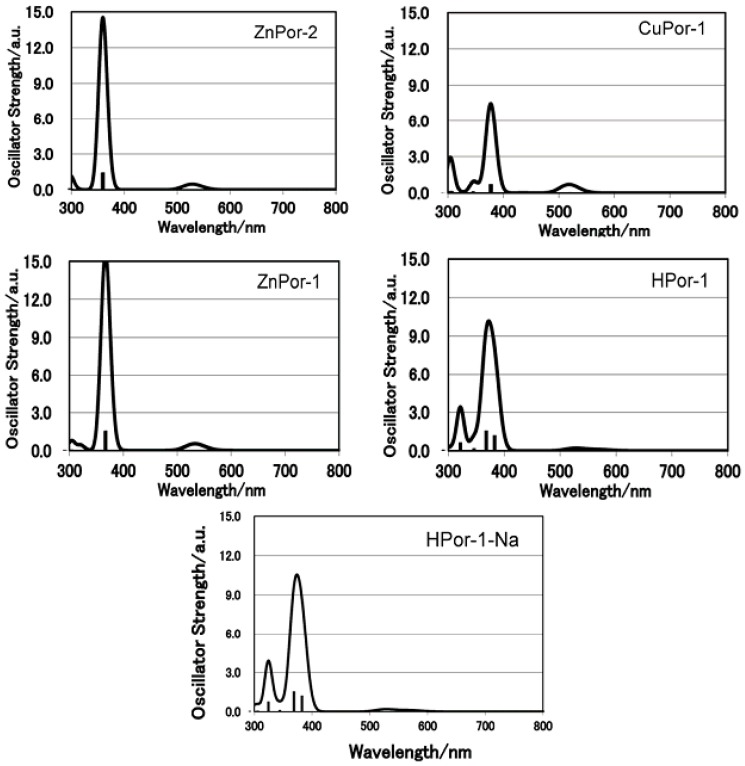
Calculated electronic absorption spectra for the set of porphyrin sensitizers.

First, the absorption capabilities of the porphyrin sensitizers were evaluated. Figure 2 shows the electronic absorption spectra of the porphyrin sensitizers dissolved in ethanol. Figure 3 shows the calculated absorption spectra with TD-DFT method in ethanol. 

The calculated absorption spectra show excellent agreement with the observed absorptions. The free-base Por-1 gives a broader absorption spectrum from 300–650 nm over these Zn- and Cu-porphyrin sensitizers. Moreover, the Q-bands of the free base Por-1 split into four absorption peaks. The fact that the absorption capability of HPor-1 is greater than that of the metallated Por-1 suggests that if the electron injection efficiencies for these sensitizers are equal, DSSCs based on the former sensitizer may generate more photocurrent than would those based on the latter sensitizers.

Replacing the protons of HPor-1 with sodium ions further shifts the Q-band absorption to the longer-wavelength region. Furthermore, the absorption spectrum of the HPor-1-Na sensitizer shows some characteristics related to dye aggregation in solution. This may be due to the reduced solubility of the dye upon deprotonation. The absorption capability of Por-1 with different central metals can be ranked as follows: HPor-1 > ZnPor-1 > CuPor-1. Meanwhile, the absorption capability of ZnPor-1, over the entire solar spectrum, is very similar to that of ZnPor-2.

**Figure 4:**
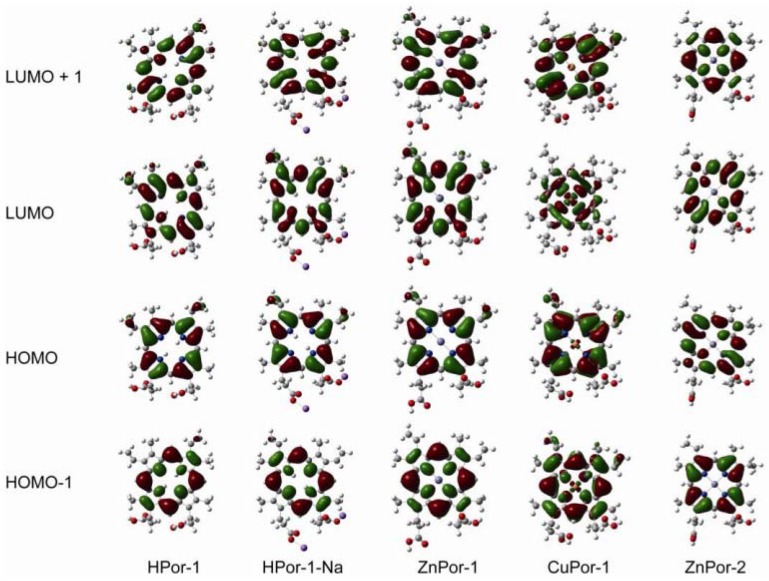
Frontier molecular orbitals of the porphyrin sensitizers based on DFT/CAM-B3LYP/6-31G (d,p) calculations with CPCM (ethanol).

To further understand the electron ejection capabilities of these porphyrin sensitizers, their molecular orbitals and energy levels are calculated by the DFT and TD-DFT methods. Figure 4 shows the molecular orbitals, including the HOMO-1, HOMO, LUMO, and LUMO+1, of the porphyrin dyes in ethanol. A comparison of these porphyrin molecules leads to the following conclusions: (1) since the density of the electron cloud at the carboxyl group is similar, the three Por-1 molecules with different central metals have similar electronic coupling abilities with semiconductors; (2) the deprotonation of HPor-1 does not affect the molecular orbital; (3) ZnPor-2 has fewer π-electrons at the β-positions of the porphyrin ring than does ZnPor-1, suggesting that the π-electrons in ZnPor-2 are more delocalized; (4) furthermore, the molecular structure of porphyrin sensitizers does not consist any electron donor groups, and intramolecular charge transfer (ICT) is not reflected by the molecular orbitals.

**Figure 5:**
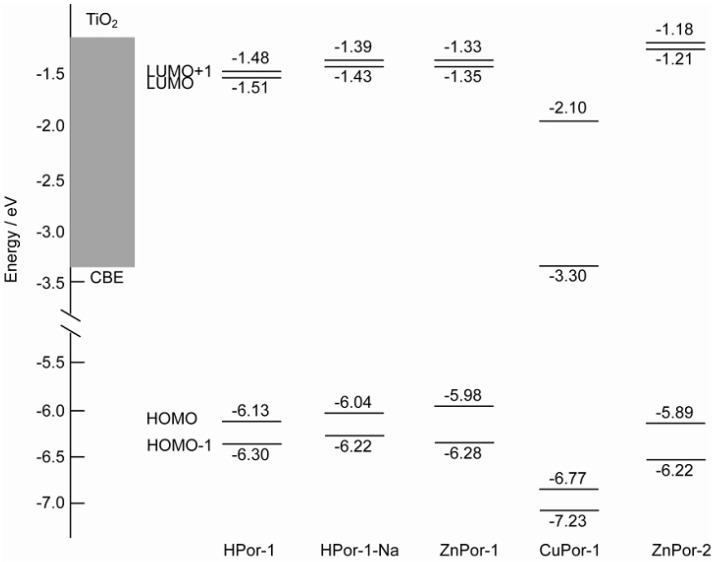
Comparison of energy levels of the HOMO-1, HOMO, LUMO, and LUMO molecular orbitals of the porphyrin sensitizers to that of the CBE of TiO_2_.

Figure 5 depicts the energy levels of the four molecular orbitals, together with the conduction band of TiO_2_. All the porphyrin sensitizers have the LUMO and LUMO+1 levels above the conduction band edge (CBE) of TiO_2_. This suggests that favorable electron injection can take place from these porphyrins into TiO_2_. However, the difference in the energy levels of the HOMO orbitals can substantially affect the photovoltaic performance, since the charge recombination kinetics are mainly determined by the difference in the CBE of the semiconductor and the HOMO level (Δ*E*_R_) of the dye. For Por-1 with different central metals, the Δ*E*_R_ values can be ranked in the following order: CuPor-1 > HPor-1 > ZnPor-1. The charge recombination will also be in the same order. Since the *V*_oc_ value of a DSSC is mainly determined by the charge recombination process, a larger Δ*E*_R_ should lead to a smaller *V*_oc_. 

On the other hand, a similar prediction may be made when comparing ZnPor-1 with ZnPor-2. Although the Δ*E*_R_ value of ZnPor-2 is larger than that of ZnPor-1, the change in the molecular structure at the β-positions can substantially affect the charge recombination process. It has been found earlier that linear alkyl groups can function as a hydrophobic shell to prevent contact between the semiconductor and the electrolyte [8]. The ethyl groups of ZnPor-2 have a similar effect, and therefore, solar cells based on ZnPor-2 should generate higher photovoltage than those based on ZnPor-1.

**Figure 6:**
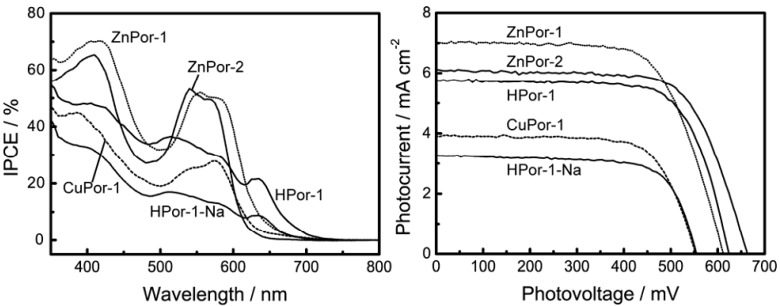
IPCE profiles and I-V curves of DSSCs based on porphyrin sensitized TiO_2_ electrodes.

The results from our experiments showed that our predictions were correct. We measured the photocurrent-photovoltage (I-V) curves and incident photon-to-current conversion efficiency (IPCE) profiles of the solar cells based on the porphyrin sensitizers (Figure 6). Table 1 lists the relevant parameters obtained from the I-V curves. For Por-1 with different central metals, the short-circuit current (*J*_sc_), open-circuit voltage (*V*_oc_), and *η* are in the following order: ZnPor-1 > HPor-1 > CuPor-1. This order is in excellent agreement with our principle shown above. The difference in the photovoltaic performances of these metal-based Por-1 sensitizers is mainly due to their different HOMO energy levels.

**Table 1:** Photovoltaic performance of DSSCs using chlorophyll derivatives having porphyrin macrocycle.

Dye sensitizer	*J*_sc_ / mA·cm^−2^	*V*_oc_ / V	FF	%
HPor-1	5.8	0.62	0.71	2.6
HPor-1-Na	3.3	0.55	0.71	1.3
CuPor-1	3.9	0.55	0.71	1.5
ZnPor-1	7.0	0.61	0.68	2.9
ZnPor-2	6.1	0.66	0.69	2.8

*J*_sc_: Short-ciruit current; *V*_oc_: open-circuit voltage; FF: Fill factor; *η*: solar energy-to-electricity conversion efficiency.

On the other hand, ZnPor-2 gives lower *J*_sc_, but higher *V*_oc_ and *η* values than does ZnPor-1 for the following reasons: the vinyl groups of ZnPor-1 extend the π-electrons out of the porphyrin core, and this improves the light-harvesting capability of this sensitizer, as shown in Figure 2 and Figure 3.The light-harvesting capability of a sensitizer is a key parameter that determines the amount of solar energy can be captured by the solar cell and thus affects the photocurrent generated by the solar cell. The higher *V*_oc_ value of the ZnPor-2-based solar cell is mainly due to the effect of the ethyl groups that prevent the leakage of electrons from TiO_2_ to the electrolyte. As a result, the overall photovoltaic performances of ZnPor-1 and ZnPore-2 are practically the same (2.9% and 2.8%, respectively).

Moreover, the deprotonated HPor-1 shows much poorer photovoltaic performance than does the pristine cell. This could be due to the formation of aggregates, which can cause serious exciton annihilation, as suggested in previous studies [7,8].

### 2.2. Chlorin Sensitizers for DSSCs

**Figure 7:**
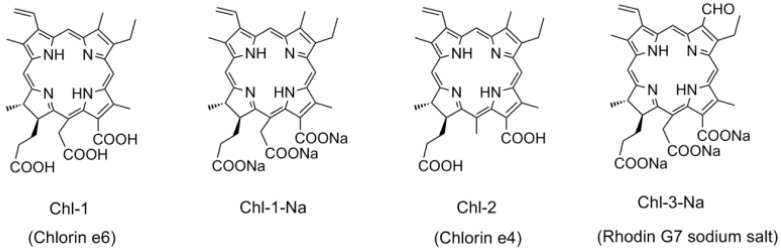
The chemical structures of chlorophyll related chlorin sensitizers.

The chlorin macrocycle forms the core structure in natural chlorophylls. This leads to chlorins having better light-harvesting capabilities than the corresponding porphyrins. Chlorin-based chlorophylls therefore are more suitable for solar cell applications. In the present study, we used chlorin sensitizers that can be easily derived from their natural chlorophyll analogs, *i.e.*, from chlorophylls *a* and *b*. Figure 7 shows the chemical structures of the chlorin sensitizers. The difference between Chl-1 and Chl-2 is in the number of carboxyl groups, *i.e.*, three for the former and two for the latter. Similar to porphyrins, Chl-1-Na can be obtained by the deprotonation of Chl-1 with sodium ions. The basic chlorin structure of both Chl-1 and Chl-2 is similar to that of their natural analog chlorophyll *a*, which has a methyl group at the C7 position. For comparison, we also tested Chl-3-Na with an aldehyde group at the C7 position, since the natural chlorophyll *b* molecule has this structure. 

**Figure 8:**
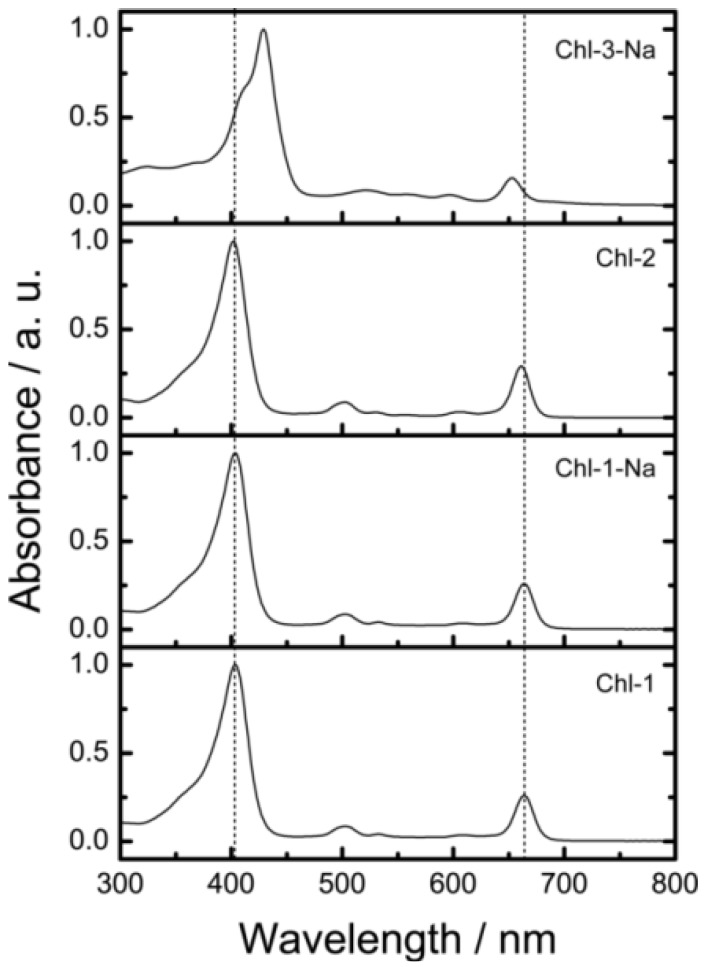
The electronic absorption spectra of chlorin sensitizers in ethanol solution.

**Figure 9:**
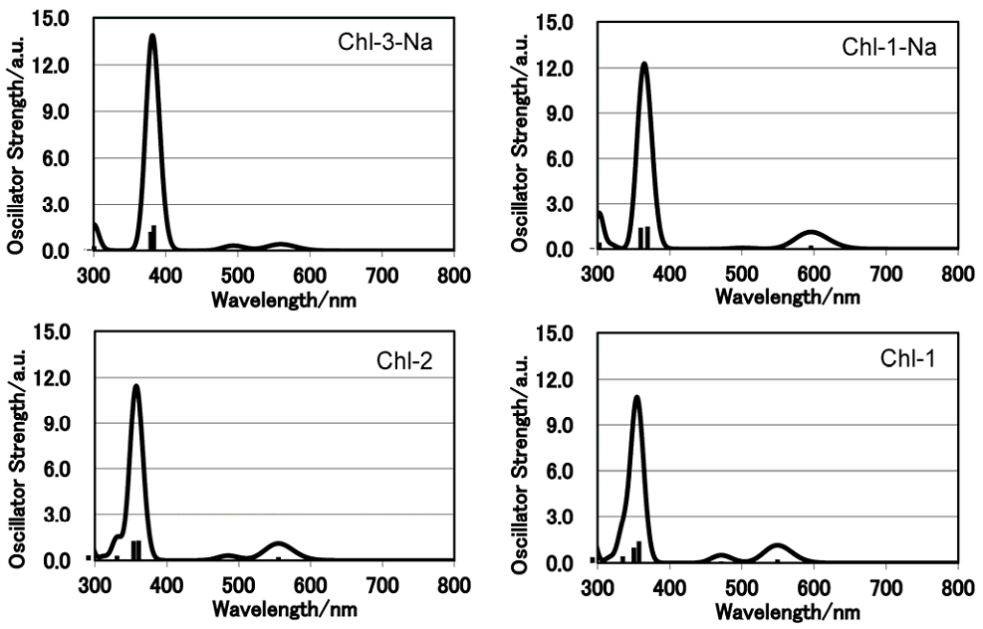
Calculated electronic absorption spectra for the set of chlorin sensitizers.

Figure 8 shows the electronic absorption spectra of the chlorin sensitizers in ethanol solution. In order to reproduce the observed absorption spectra with molecular simulation, Figure 9 shows the calculated absorption spectra with TD-DFT method in ethanol. 

It is clear that a change in either the number of carboxyl groups or the number of protons does not strongly affect the absorption spectrum. The Chl-1, Chl-2, and Chl-1-Na sensitizers have identical absorption patterns. This phenomenon is different from that observed for porphyrin sensitizers. In the case of porphyrin sensitizers, the deprotonated porphyrins aggregate in solution. In addition, the absorption region of the Chl-3-Na synthesizer is narrower than that of the Chl-1-Na sensitizer.

**Figure 10:**
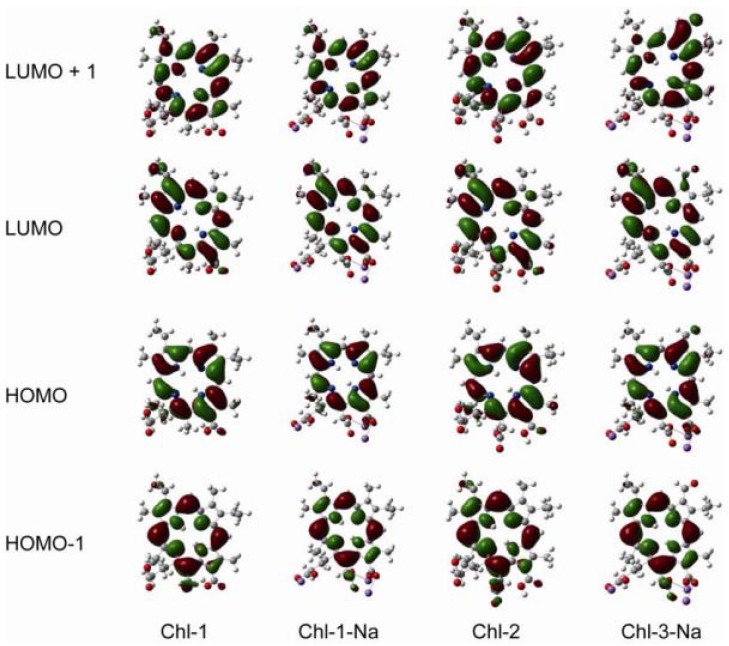
Frontier molecular orbitals of the chlorin sensitizers based on DFT/CAM-B3LYP/6-31G (d,p) calculations with CPCM (ethanol).

DFT calculations are performed for the chlorin sensitizers as well. Figure 10 shows the frontier molecular orbitals of the chlorin sensitizers in ethanol solution. The molecular orbitals of Chl-1, Chl-1-Na, and Chl-2 are very similar to each other. This is because a change in the unconjugated carboxyl groups does not alter the distribution of electrons over the cyclic tetrapyrrole ring. However, because of the aldehyde group at the C7 position, the LUMO of Chl-3-Na shows extended electrons in the direction of the Q_x_ transition dipole.

**Figure 11:**
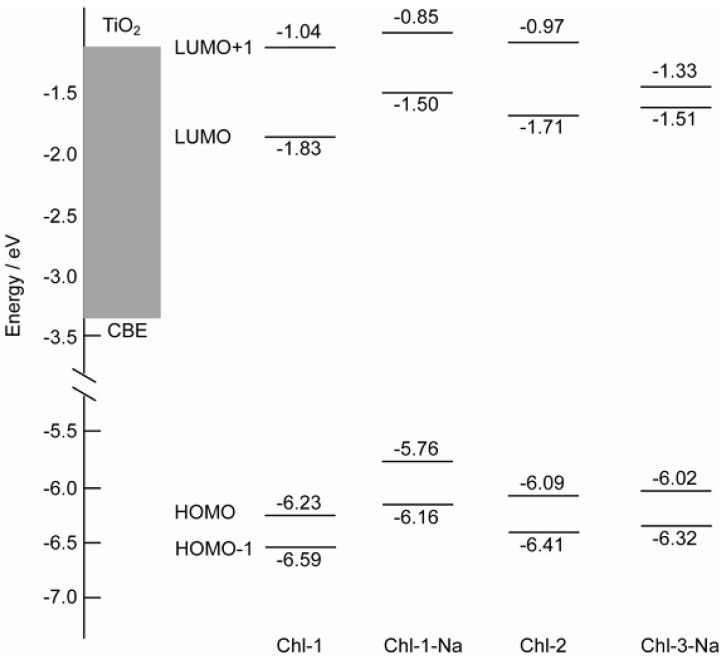
Comparison of energy levels of the HOMO-1, HOMO, LUMO, and LUMO molecular orbitals of the chlorin sensitizers to that of the CBE of TiO_2_.

The energy levels of the four molecular orbitals have been compared in Figure 11. The energy levels of the LUMO of the chlorin sensitizers are also sufficiently higher than the CBE of TiO_2_. Thus, a major factor that determines the photovoltaic performance must be related to the charge recombination process rather than to the electron injection process. Similar to porphyrin dyes, charge recombination should be more pronounced in chlorin sensitizers with a deeper HOMO energy level. Chl-1, with the deepest HOMO level, seems to be the best suited for the reverse electron transfer process and thus should give the lowest *V*_oc_ and *η* values.

**Figure 12:**
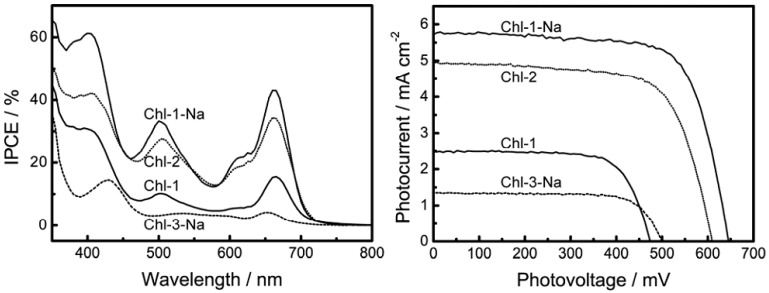
IPCE profiles and I-V curves of DSSCs based on chlorin sensitized TiO_2_ electrodes.

In order to test the theory stated above regarding HOMO levels to determine the photovoltaic performance, we fabricated DSSCs based on the chlorin sensitizers. Figure 12 shows the I-V curves and IPCE profiles of DSSCs based on the chlorin sensitizers, and Table 2 lists the relevant parameters obtained from the I-V curves. In contrast to its effect on the porphyrin sensitizers, deprotonation of the Chl-1 sensitizer substantially improves the photovoltaic performance. Except in the case of Chl-3, which has a different chlorin frame, the *J*_sc_, *V*_oc_ and *η* values were 2.5 mA·cm^−2^, 0.47 V, and 0.9% for Chl-1, 4.9 mA·cm^−2^, 0.61 V, and 2.1% for Chl-2, and 5.7 mA·cm^−2^, 0.65 V, and 2.7% for Chl-1-Na. This effect of protons can be attributed to the change in the HOMO energy levels, as predicted in the previous section.

**Table 2:** Photovoltaic performance of DSSCs using chlorophyll derivatives having chlorin macrocycle.

Dye sensitizer	*J*_sc_ / mA·cm^−2^	*V*_oc_ / V	FF	%
Chl-1	2.5	0.47	0.72	0.9
Chl-1-Na	5.7	0.65	0.72	2.7
Chl-2	4.9	0.61	0.69	2.1
Chl-3-Na	1.4	0.50	0.73	0.5

*J*_sc_: Short-ciruit current; *V*_oc_: open-circuit voltage; FF: Fill factor; *η*: solar energy-to-electricity conversion efficiency.

The poorer photovoltaic performance of Chl-3-Na as compared to that of Chl-1-Na is attributable to the following reasons: (1) the light-harvesting capability of Chl-3-Na is the lowest among that of all the chlorin sensitizers; (2) the HOMO level of this dye is favorable for charge recombination; and (3) most importantly, the LUMO+1 and LUMO of this particular dye are close to each other, making the excitation relaxation too slow to facilitate electron injection from the LUMO level.

We also critically examined the porphyrin and chlorin sensitizers as potential candidates for real-world applications. The photovoltaic performance of the best sensitizer studied in the present investigation is still lower than that of the state-of-art sensitizers currently used in DSSCs. However, the sensitizers that we studied are much more inexpensive and environment-friendly. Moreover, the photovoltaic performance of these commercially available dyes might be improved by selective co-sensitization [13].

## 3. Experimental

### 3.1. Dye-Sensitizers

Dye sensitizers were obtained from Frontier Scientific (Logan, UT, USA) and used as received.

### 3.2. Fabrication of Dye-Sensitized Solar Cell and Photovoltaic Measurements

The OTE with 0.25 cm^2^ working area contains 18 nm and 400 nm TiO_2_ nanoparticles with thickness of 10 μm and 4 μm obtained from CCIC, for light-harvesting and light scattering, respectively. The details of fabrication of DSSCs were described before [14]. Each DSSC that was fabricated by the use of this OTE, the counter electrode of Pt-sputtered FTO glass (10 Ω·cm^−2^, Nippon Sheet Glass, Osaka, Japan), and the electrolyte A containing 0.1 M LiI, 0.05 M I_2_, and 0.6 M 1-propyl-3-methylimidazolium iodide in a mixture of acetonitrile and valeronitrile (1:1, v/v). The experimental set-up for measurements of I-V characteristics and IPCE profiles was described before [14].

### 3.3. DFT and TD-DFT Calculations

All systems were optimized by the use of DFT calculation with the CAM-B3LYP [15,16,17] exchange-correlations functional and the 6-31G(d,p) [18] basis set, and with solvation effect for the free base sensitizer described by CPCM (ethanol) [19]. The TD-DFT [20] with the CAM-B3LYP exchange-correlations functional and the 6-31G(d,p) basis set calculations are based on the optimized structure with solvation effect described by CPCM (ethanol) for the set of sensitizers. All calculations were done by Gaussian 09 [21] using the Research Center for Computational Science, Okazaki, Japan.

## 4. Conclusions

In the present study, natural-chlorophyll-related porphyrins and chlorins have been used as dye sensitizers for DSSCs. In the porphyrin sensitizers, the central metal can substantially affect the photovoltaic performance. Among 2H-, Cu-, and Zn-based porphyrin sensitizers, the Zn-based porphyrin sensitizer gives the highest conversion efficiency, which can be up to 2.9%. Deprotonation of porphyrin sensitizers reduces the overall conversion efficiency. This is because the dye molecules readily aggregate in solution. In contrast, the deprotonation of chlorin-based sensitizers can substantially improve their photovoltaic performance. The energy level of the HOMO of the dye sensitizers is considered to have the strongest effect on the photovoltaic performance. This is because of the kinetics of charge recombination between TiO_2_ and the dye sensitizer. 
